# Congenital Anophthalmia: A Review of Dealing with Volume

**DOI:** 10.4103/0974-9233.63082

**Published:** 2010

**Authors:** C. Robert Bernardino

**Affiliations:** Section of Ophthalmic Plastics and Orbital Surgery, Department of Ophthalmology and Visual Science, Yale School of Medicine, New Haven, CT, USA

**Keywords:** Anophthalmia, Congenital, Expander, Hydrogel, Microphthalmos, Orbit

## Abstract

**Background:**

Anophthalmia in childhood whether congenital or acquired is not just a question of cosmesis. Loss of an eye can effect the maturation of the soft tissues and bony structure surrounding the affected orbit. Therefore, a comprehensive approach including medical and surgical interventions is required to rehabilitate a child early in life.

**Materials and Methods:**

A literature survey of the past 40 years on the topic of congenital anophthalmia with focus on medical and surgical volume augmentation of the orbit was conducted.

**Results:**

Newer technologies including hydrogel implants and saline-filled tissue expanders have allowed for more rapid expansion of the pediatric orbit often with minimally invasive surgical procedures. However, traditional approaches including conformer therapy are still the primary intervention in these complicated cases.

**Conclusion:**

Anophthalmia in childhood requires a close interaction between ophthalmologist and ocularist as well as a motivated patient and family. With early intervention a good cosmetic outcome with periocular symmetry is obtainable.

## INTRODUCTION

**Figure 1:**
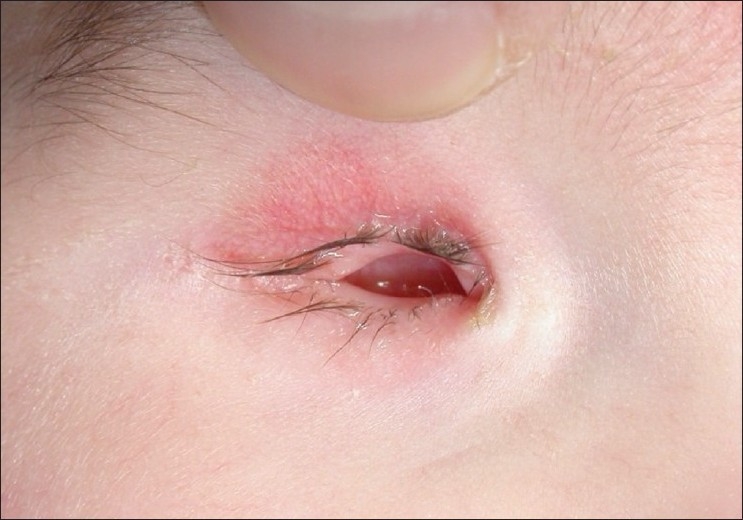
External photograph of a 1-year child with congenital anophthalmia of right side. Note the normal, albeit small eyelids and hollow eye socket

**Figure 2:**
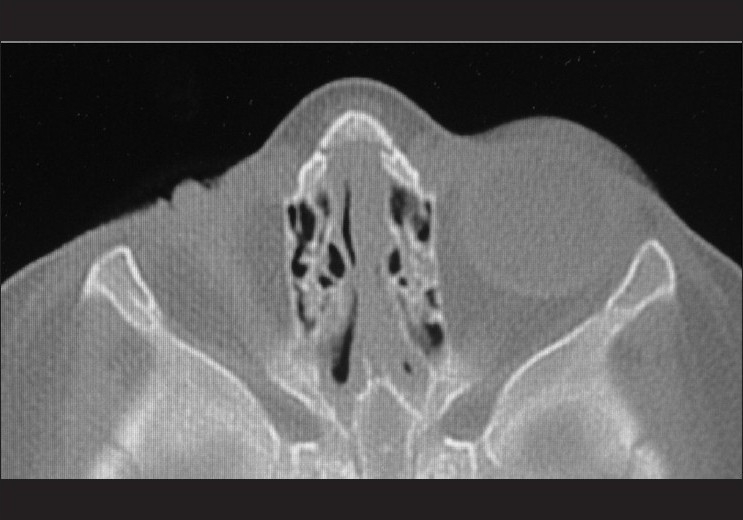
Computerized axial tomography (CAT) scan, bone window, coronal cut of the same patient in Figure 1. Note the poorly formed remnant of globe and hypoplastic bone structure of the right socket compared to the left

Maldevelopment of an eye or loss of an eye early in life has many ramifications including monocular status, aesthetics, and the psychosocial challenges for a child. These are not unique compared to the loss of an eye in adulthood. However, what makes loss of an eye early in life unique is the loss of volume of the eye which can lead to hypoplasia of the orbit and surrounding face. In particular, both the soft tissue of the periocular region including eyelids and eye socket, as well as the bony orbit and midface can be hypoplastic [Figures 1 and 2]. Therefore, early identification and intervention are the key factors. This article reviews the many different methods to improve orbital volume after loss of an eye in childhood.

## CLINICAL EVALUATION

When choosing a modality to treat an anophthalmic socket, one must consider the cause of the loss, status of the affected eye, the contralateral eye, the severity of volume deficit, the age of the child, and psychosocial issues including the support system of the child and the maturity of the child.^1^ If the affected side has a seeing microphthalmic eye, options are somewhat limited, as most interventions can adversely affect the vision. A clear conformer treatment is ideal, since vision can be preserved in the seeing eye. Once a painted conformer is desired, the pupil can be left clear to allow for continued visual development.

If the patient has a normal contralateral side, then therapy should be initiated early, because asymmetry can be pronounced. Furthermore, volume deficit may be significant, and therefore treatment will need to address this specifically. However, if both sides are affected either with bilateral microphthalmia or anophthalmia, asymmetry may be minimal and not noticeable. Therefore aggressive therapy may not be required.

The loss of the eye can also determine treatment options. If the child had the eye removed for treatment of a malignant lesion in the setting radiotherapy, some treatments such as dermis fat grafting may not be successful due to radiotherapy.

Addressing psychosocial issues are key in the successful treatment of the congenital anophthalmic patients. Cooperation and active participation of the patient's parents are essential, particularly for conformer therapy. Surgical interventions require less parental participation, but the burden of multiple surgeries and prolonged recovery requires parental support. These are important considerations especially when the child is school aged. Finally, frank discussion with the family as to realistic outcome of therapy is vital.

**Figure 3:**
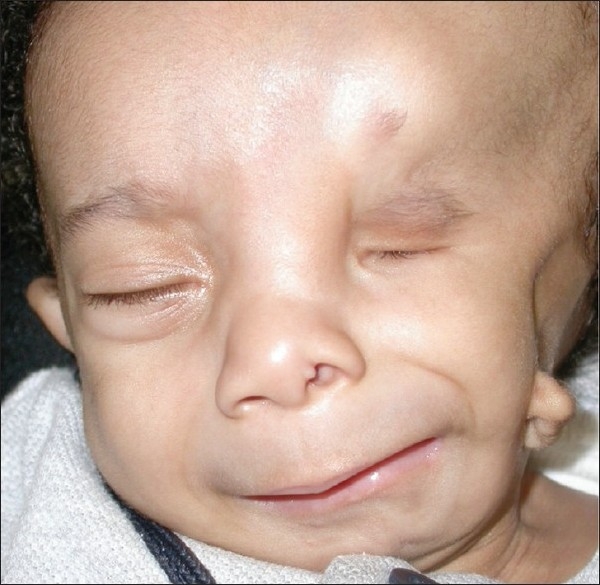
External photograph of a 6-month-old child with severe Goldenhar syndrome, including microphthalmia, midface and lower face hypoplasia, microtia

Besides a focus on volume asymmetry, the status of the periocular structures needs to be addressed. This should include evaluation of the eyelids, eyebrows, midface, forehead, and even the lower face and ears. Often anophthalmia or microphthalmia is associated with other congenital maldevelopment including hypoplasia of the midface, microtia, or hypoplastic jaw and mouth [Figure 3]. Addressing the orbital volume often deals with the other problems. However, coordinating intervention for these problems and including needs for medical and surgical interventions as well as timing is a must among multiple caregivers.

## DIAGNOSTIC STUDIES

Orbital imaging is very helpful in determining both the status of the eye, whether there are associated with periocular and intracranial abnormalities, and the degree of orbital hypoplasia. However, each imaging modality has limitations particularly in the pediatric population.

Orbital ultrasound is helpful during initial evaluation of the patient, particularly to evaluate the status of the eye to determine whether it is microphthalmic or truly anophthalmic. It can be performed in the clinic without the administration of anesthesia. However, it cannot image the bony orbit or other periocular structures. It is a quick imaging intervention and therefore is probably the primary imaging modality before any other imaging.

Magnetic resonance imaging (MRI) may be the second imaging study ordered. This is because it can image the soft tissues of the orbit and brain, and does not have any side effects on the children. Usually, a pediatrician or geneticist would like to obtain an MRI image of the brain to ensure the absence associated intracranial abnormalities. MRI, however, requires sedation or general anesthesia depending on the child, and therefore is not readily obtained. Furthermore, the bony anatomy around the eye is not directly imaged, but can be inferred. Therefore, MRI may be an initial imaging study after orbital ultrasound, and can also be used for follow-up after medical and surgical interventions.

Computerized axial tomography (CAT) scan is the most useful for physicians interested in surgical interventions around a hypoplastic orbit. It allows for imaging of the bone including three-dimensional reconstructions; based on these scans a plastic model can even be constructed of the patient's skull for presurgical planning. CAT scans are also rapid and therefore a child can be imaged often with just sedation alone. However, the significant amounts of radiation exposure to children is a concern because multiple CAT scans put a child at risk for radiation malignancies which can be as high as 1 in 1000.^2^ This risk can be minimized using parameters appropriate for children that decreases the exposure. Most children requiring surgical intervention will need a presurgical CAT scan. Follow-up imaging can be performed by MRI, required to limit radiation exposure.

## MEDICAL MANAGEMENT

**Figure 4:**
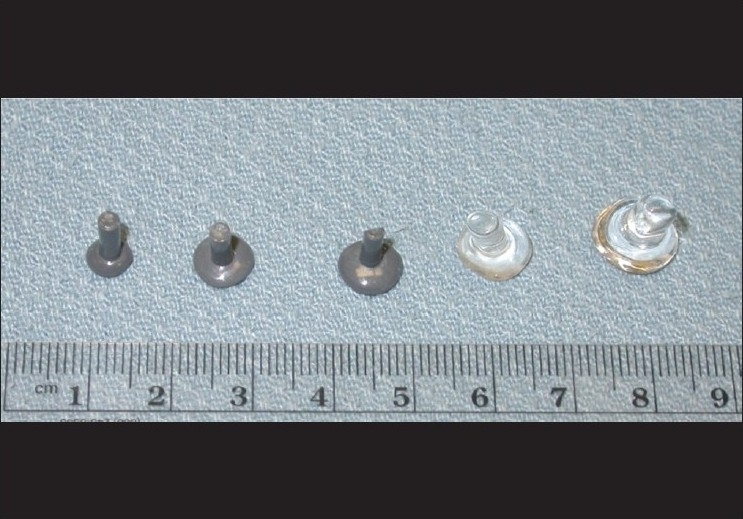
Serial conformers used to expand socket of patient in Figure 1

There are a number of methods to stimulate orbital volume development, both medical and surgical. The main medical method is serial conformer fittings.^3^ In conjunction with an ocularist, a conformer is made to fit the space behind the eyelids. These can be extremely small if there is no socket at all. Then as the socket conforms, stretches, and grow to accommodate the conformer, a larger conformer is made to replace the previous one [Figure 4]. This serial augmentation takes time, and parent cooperation is key. If a conformer is displaced, the parents need to replace it. If a conformer is left out for significant period of time, contraction and reversal of benefit often occur. A conformer can be worn in a completely anophthalmic socket that lacks an orbital implant, over a socket with an implant, and over a microphthalmic eye. Conformers can even be fashioned in the setting of microblepharon.^4^ Therefore, it is quite versatile. Usually, the conformer is left clear and a small peg or handle can be placed centrally to help with handling, particularly for parents. However, it can also be painted if desired and the pupil can even be left clear if an underlying microphthalmic eye has vision.

## SURGICAL MANAGEMENT

Surgical intervention involves placing a space occupying implant or device to encourage orbital growth. An ideal implant would be easy to place, completely biocompatible, and volume titratable over time. However, current surgical modalities are compromises of the ideal implant. Surgical interventions are divided into two categories: static and dynamic. Static means that the volume implant does not change volume over time, whereas dynamic means that the volume can increase over time. Static is a very traditional approach. A spherical implant, typically smooth acrylic or silicone, is placed into the eye socket.^5^ Some surgeons choose to start with a small implant and perform serial surgeries every year or two and slowly increase the size of the implant.^6^ The advantage of this approach is that it mimics orbital development to some degree. However, it exposes the child to numerous surgeries. The other method chosen is to place as large an implant as possible, regardless of the amount of orbital hypoplasia present. This lessens the number of surgical interventions required but places the patient at risk of implant exposure or extrusion.

Dynamic orbital implants are advantageous compared to static in that volume of the implant increases to stimulate orbital growth even further. One of the most traditional approaches to eye socket reconstruction, dermis fat grafting, is a dynamic implant.^7^ Dermis fat grafts in many ways are the ideal implant; they are biocompatible and grow slowly over time. However, dermis fat grafting is not without limitations. The graft requires harvesting which means there is a second surgical site, typically the buttocks, which in turn leads to a scar. The graft compatibility and growth can be variable; the fat can atrophy or in rare cases hypertrophy can occur necessitating debulking. Dermis fat grafts also take time to heal, and during this period discharge, bleeding, or pyogenic granuloma can occur. Despite these limitations and a learning curve, this a very good option to address the hypoplastic orbit in congenital anophthalmia.

Other dynamic implants involve either a fluid chamber in which saline is injected into the chamber and chamber expands accordingly, or a synthetic material which expands over time. The saline chamber is essentially a modification of the traditional tissue expander. It usually has two components, the bladder and the injection port. The bladder is spherical in configuration and needs to be fixated to bone; it is usually placed in the subperiosteal space of the orbit. The filling port is similar to subcutaneous ports used for chemotherapy. It is usually placed in the temporalis fossa. A filling tube is tunneled and pasted through the lateral orbital wall to connect to the bladder. Once in place, injections of saline are placed in the subcutaneous port and the chamber expands.^8^ This allows for titration of the volume. However, injections can be painful for the patient, and the chamber can exert significant pressure which can lead to erosions or extrusion. This pressure can also cause atrophy of the surrounding tissue. Once the desired volume is met, the port and bladder need to be removed and replaced with a permanent implant. These saline-filled tissue expanders have a long track record, and therefore many surgeons are comfortable using them.

More recently, solid hydrophilic tissue expanders have been designed for orbit expansion.^9^ Created out of hydrogel (methylmethacrylate and *N*-vinylpyrrolidone), they are the same material as contact lens and scleral buckles. These expanders when dehydrated, they are small and solid and when hydrated, they expand slowly to a final volume which is similar to gelatin. Since they are small when dry, they can be inserted through a small incision; this allows for shorter surgery time and faster recovery. Furthermore, since the hydrogel expands slowly, the risk of associated tissue atrophy and exposure or extrusion is theoretically lower. This material has been used in similar instances as the more traditional saline tissue expander, and is now fashioned for orbital use both as a hemisphere and a sphere.^10^

**Figure 5:**
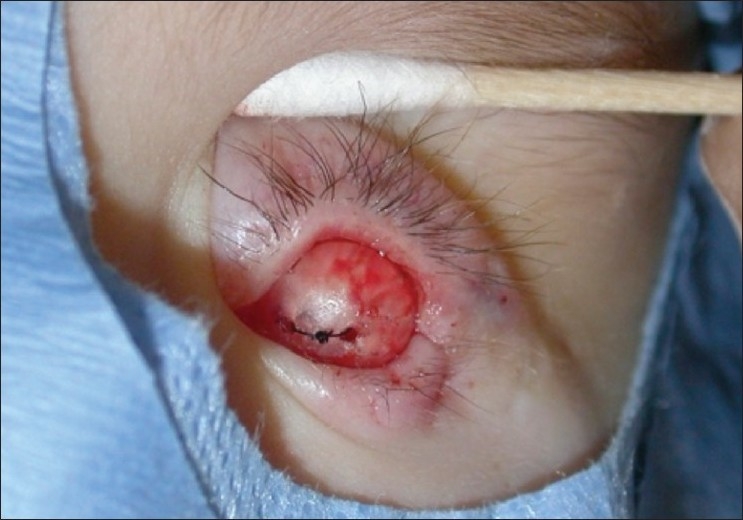
Hydrogel hemisphere sewn in to conjunctival socket

**Figure 6:**
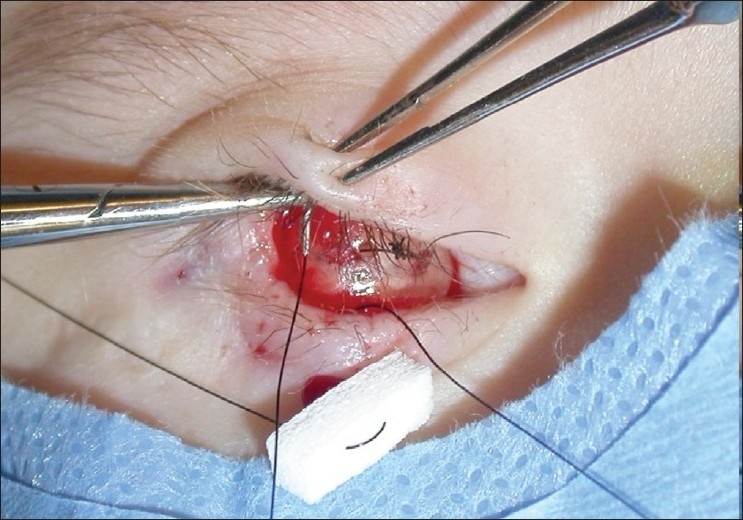
Suture tarsorrhaphy over hemisphere expander

The hemisphere is used to expand the conjunctival socket. It is available in three sizes: 0.4cc, 0.9cc, and 1.5 cc. It is sewn into the conjunctival space [Figure 5], and the eyelids are sewn over top with a suture tarsorrhaphy [Figure 6]. According to the author, these are left in place for a month and then either upsized or replaced with solid conformer. If the hemisphere is oversized, the tarsorrhaphy can prematurely breakdown. Furthermore, the eyelids can become inflamed and uncomfortable, or pyogenic granuloma can form. However, it is quite effective, particularly when development has stalled with conformer therapy or the family/child cannot cooperate with conformer therapy.

Once the conjunctival socket is expanded, orbit expansion can commence with spherical hydrogel implants. These are available in the volumes of 2.0cc, 3.0cc, and 4.0 cc volumes. A lateral canthal incision is made, and subperiosteal dissection is performed. The periosteum is incised deep in the orbit, and the dehydrated sphere is inserted into the deep orbit. A transconjunctival approach can also be used with similar results. Since the resulting expanded implant is still solid in nature, it can be left in the orbit long term. If removal is required, it is removed piece meal due to its gelatin-like consistency. One concern of long term use is whether they truly stop expanding; hydrogel scleral buckles have been found to over expand over time. The chemical structure differs perhaps negating this possibility, but long term follow-up is necessary to address the possibility of continued expansion.

**Figure 7:**
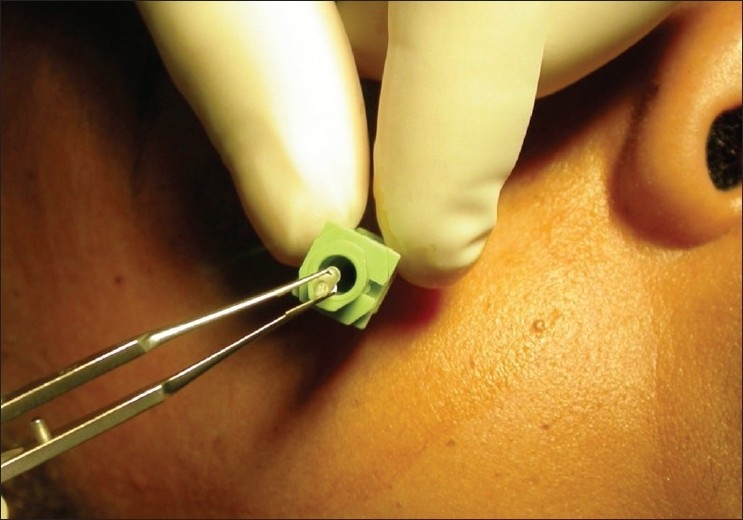
Trochar inserted transcutaneously at the inferior orbital rim. Hydrogel pellet (0.2 cm^3^) is inserted into trochar and deposited into the posterior orbit. This photo is an example in an adult anophthalmic patient

A final form of these hydrogel implants is an injectable pellet. Each pellet is 0.2 cm^3^ in final volume, but can be injected through a small trochar. Injection can be administered under local anesthesia in adults or mask anesthesia in the child. The trochar is inserted transcutaneously at the inferior orbital rim and directed into the deep orbit [Figure 7]. The trochar site does not require suturing. The advantage of the ijuctable pallet is that volume can be titrated overtime. There is a risk of pellet extrusion through the trochar tract, and injecting more than 1.0cc of volume at a time can be very painful. However, Schittkowski has reported succes in the treatment of congenital anophthalmia.^11^ These pellets approach the ideal expander because they are biocompatible, easy to insert, and titratable. They can even be used behind the nonseeing microphthalmic eye; however, use behind the microphthalmic eye with vision is not advisable.

A final approach to treating the hypoplastic orbit is orbito-cranial advancement surgery. This involves multiple osteotomies to release the periocular bones from the orbit, and advancing them forward and outward with bone grafts and plating.^12^ These are usually reserved when other techniques fail, or if a patient presents late necessitating aggressive therapy.

## FOLLOW-UP

After any intervention, close follow-up is important. Patients must be monitored closely for response to orbit volume expansion therapy. In particular, any therapy which requires parent participation, such as conformer therapy, multiple visits are vital not only to monitor progress, but also to encourage compliance with therapy. Reassessing functional and cosmetic goals with the parents is also important to keep all caregivers focused on the end point and the time frame to reach that endpoint. Finally being mindful of social time frames, the patient entering school for example, will provide a realistic frame of reference for interventions and outcomes.

In conclusion, congenital anophthalmia is one of the more challenging scenarios to manage. It requires a comprehensive approach with multiple caregivers including the ophthalmologist, ocularist, pediatrician, and the patient's family.^13^ However, early intervention can lead to very satisfactory results allowing for normal anatomic and psychosocial development.
